# Familial Mediterranean Fever as a Cause of Recurrent Pleurisy in a Child With Crohn's Disease: A Case Report

**DOI:** 10.1155/crpu/9412773

**Published:** 2025-07-21

**Authors:** Ola Alhalabi, Khaled Abouhazima, Fatima Al Maadid, Ahmed Abushahin

**Affiliations:** ^1^Department of Pediatric Pulmonology, Sidra Medicine, Doha, Qatar; ^2^Department of Pediatric Gastroenterology, Sidra Medicine, Doha, Qatar; ^3^Department of Pediatric Medicine, Sidra Medicine, Doha, Qatar

**Keywords:** Crohn's disease, familial Mediterranean fever, IBD, inflammation, pleuritis

## Abstract

**Introduction:** Familial Mediterranean fever (FMF) and Crohn's disease (CD) are chronic autoinflammatory disorders that share similar clinical and biological features. Both disorders are characterized by chronic and relapsing symptoms. In some cases, FMF can coexist with CD, which makes diagnosis and treatment challenging.

**Case Presentation:** A 9-year-old Egyptian child was diagnosed with CD at the age of 5, based on clinical symptoms and endoscopic histopathologic findings. Initially, the patient responded well to biological therapy (anti-TNF*α*), showing improvements in symptoms along with declines in inflammatory markers and fecal calprotectin levels. At the age of 7, the child began experiencing frequent episodes of fever accompanied by pleuritic chest pain. Right-sided pleural effusion was identified on repeated chest X-rays. The patient was diagnosed with recurrent acute bacterial pneumonia due to immune suppression and was managed with multiple courses of oral antibiotics. During the last presentation, in one of these episodes, further investigation was pursued due to a lack of response to antibiotics, indicated by persistently elevated inflammatory markers (CRP, ESR) and nonresolving mild right-sided pleural effusion. FMF was suspected based on the patient's clinical presentation and chest imaging. A detailed family history revealed a positive FMF diagnosis in a first-degree cousin. Genetic testing was performed, which revealed two heterozygous pathogenic mutations that support the FMF diagnosis. Treatment with colchicine prevented further episodes. The patient continued to receive follow-up care from the GI team for CD and was maintained on adalimumab.

**Conclusions:** FMF should be considered for children with CD who exhibit pulmonary symptoms that do not respond to CD treatment.

## 1. Introduction

Crohn's disease (CD) is a type of inflammatory bowel disease (IBD) caused by an abnormal immune response to intestinal antigens, representing a complex interplay of genetic, immune, and environmental factors [1]. CD primarily affects the small intestine, but extraintestinal manifestations involving the joints, liver, and lungs are not uncommon. The diagnosis of CD can be challenging due to its overlap with other autoimmune diseases, which is also true for many other autoimmune diseases. Familial Mediterranean fever (FMF) is one such disease that can overlap with CD. FMF is a rare monogenic autoinflammatory disease characterized by recurrent fever, abdominal pain, and serositis. Serositis may present as pleuritis, peritonitis, or monoarthritis [2, 3].

FMF is caused by mutations in the MEFV gene, located on Chromosome 16p13. This gene encodes a protein called pyrin, which plays a role in the innate immune response. Pyrin also interacts with the *NLRP3 protein*, which has recently been identified as a risk factor for CD [4].

FMF affects over 100,000 people per year worldwide and is most prevalent among populations in the Mediterranean region, particularly Turks and Arabs [4]. Studies have indicated that the incidence of IBD is also high in this region [5]. There is a notable frequency of concomitant IBD and FMF in the same patients. One study revealed that eight (3.8%) out of 210 patients with CD have FMF, which is significantly higher than the expected rate in the FMF-endemic area [5]. Another study discovered that 15.4% of patients with FMF have concomitant IBD [6]. These studies suggest a potential shared disease mechanism between the two conditions.

This strong link between CD and FMF is also related to symptom overlap. Both FMF and CD have remission and relapse cycles, affecting the same organ systems, including the digestive tract, skin, lungs, and joints. This overlap makes the diagnosis of CD, FMF, or both sometimes challenging [2, 7]. Therefore, it is important for clinicians to recognize the strong association between CD and FMF to reach an accurate diagnosis and provide appropriate management. We present a case of a child with both CD and FMF who experienced symptoms for a few years before a definitive diagnosis of concomitant FMF could be established.

## 2. Case Presentation

**Figure 1:**
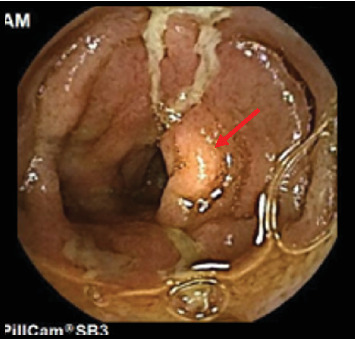
Capsule endoscopy revealed severe ulceration and exudate in the duodenum, indicated by the red arrow.

The patient is a 9-year-old Egyptian boy with a medical history significant for being diagnosed with CD at the age of 5 after presenting with chronic abdominal pain, microcytic anemia, and poor weight gain. An extensive evaluation by the gastroenterology team at that time led to the diagnosis of CD, confirmed both macroscopically and microscopically. Genetic testing for early-onset IBD was negative. Serological inflammatory markers were elevated, and fecal calprotectin levels were high (734 mcg/g). Capsule endoscopy revealed areas of inflammation in the distal duodenum and terminal ileum, supporting the diagnosis of CD (Figure 1).

Shortly after the diagnosis was made, he was started on anti-TNF*α* biologics (adalimumab), which led to a significant improvement in symptoms, weight gain, and a remarkable reduction in inflammatory markers and fecal calprotectin.

At 7 years old, the patient began experiencing clusters of febrile illnesses accompanied by chest pain. Each episode lasted 2–3 days, with complete resolution of symptoms in between. The chest pain was right-sided and worsened during inspiration. In the past 2 years, he experienced a total of seven episodes. During these episodes, there was no history of chronic cough, sputum production, or breathing difficulties. Additionally, there was no history of night sweats or weight loss. There were no reports of abdominal pain, diarrhea, or blood per rectum. Multiple chest X-rays were performed, which repeatedly showed a small right-sided pleural effusion without lung infiltrates or hilar lymphadenopathy. During these episodes, he was diagnosed with acute pneumonia and received treatment with oral antibiotics. The recurrent infections were believed to be due to immune suppression.

During the fifth episode, the patient was admitted for further investigation. He was afebrile upon admission. His respiratory rate was elevated at 35 breaths per minute, but he showed no signs of respiratory distress. Oxygen saturation was normal in room air. Chest examination revealed reduced air entry in the right lower lung fields without any adventitious sounds. The abdomen was soft, nontender, and nondistended, with normal bowel sounds. The neurological examination showed no abnormalities. There were no skin rashes and no swollen or red joints. The rest of the examination was otherwise normal.

Blood testing revealed the following: WBC count was 15.2 × 10^9^/L, Hb was 120 g/L, and platelet count was  234 × 10^9^/L. Inflammatory markers were elevated with serum CRP of 157.0 mg/L and ESR of 48 mm/h. Blood cultures were negative. ANA and double-stranded DNA antibodies were also negative. Respiratory virus polymerase chain reaction (PCR) panel and QuantiFERON test were also negative. Fecal calprotectin level was normal at 41.50 mcg/g.

**Figure 2:**
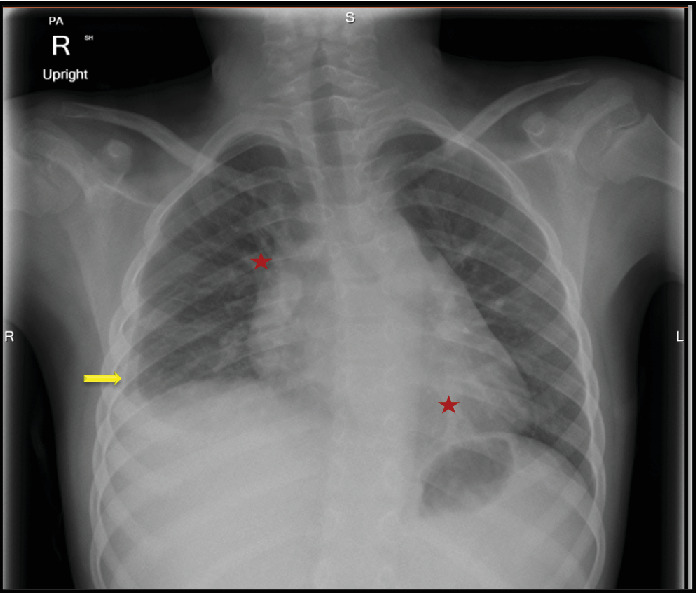
The PA view of the chest X-ray revealed decreased volume in the right lung, accompanied by mild perihilar pulmonary congestion and reticular opacification (red stars). The left lung appears adequately expanded, exhibiting subsegmental streaky atelectasis in the left retrocardiac area (red stars). Mild right basal and lamellar pleural effusions obliterating the right costophrenic angle (yellow arrow) were observed.

Repeat chest X-ray revealed the presence of pleural effusion on the right side (Figure 2). Chest ultrasound confirmed the presence of small nonloculated right-sided pleural effusion. Echocardiogram showed no pericardial effusion. Chest CT scan revealed nodular pleural thickening and small pleural effusion on the right side with no signs of parenchymal lung disease.

Aspirating the pleural fluid by thoracentesis was attempted, but only a small amount of clear fluid was collected. TB PCR, bacterial PCR, and culture of the pleural fluid were all negative. The pleural fluid smear revealed the presence of multinucleate giant cells.

Based on the clinical presentation, chest imaging, and the patient's ethnic background, FMF was suspected. Upon further questioning, the family disclosed FMF in one of the patient's first-degree cousins. Genetic testing for FMF-associated mutations was performed using PCR and reverse hybridization techniques, revealing heterozygous pathogenic mutations in the *MEFV* gene, namely *M680I (G/A)* and *M694I*.

**Figure 3:**
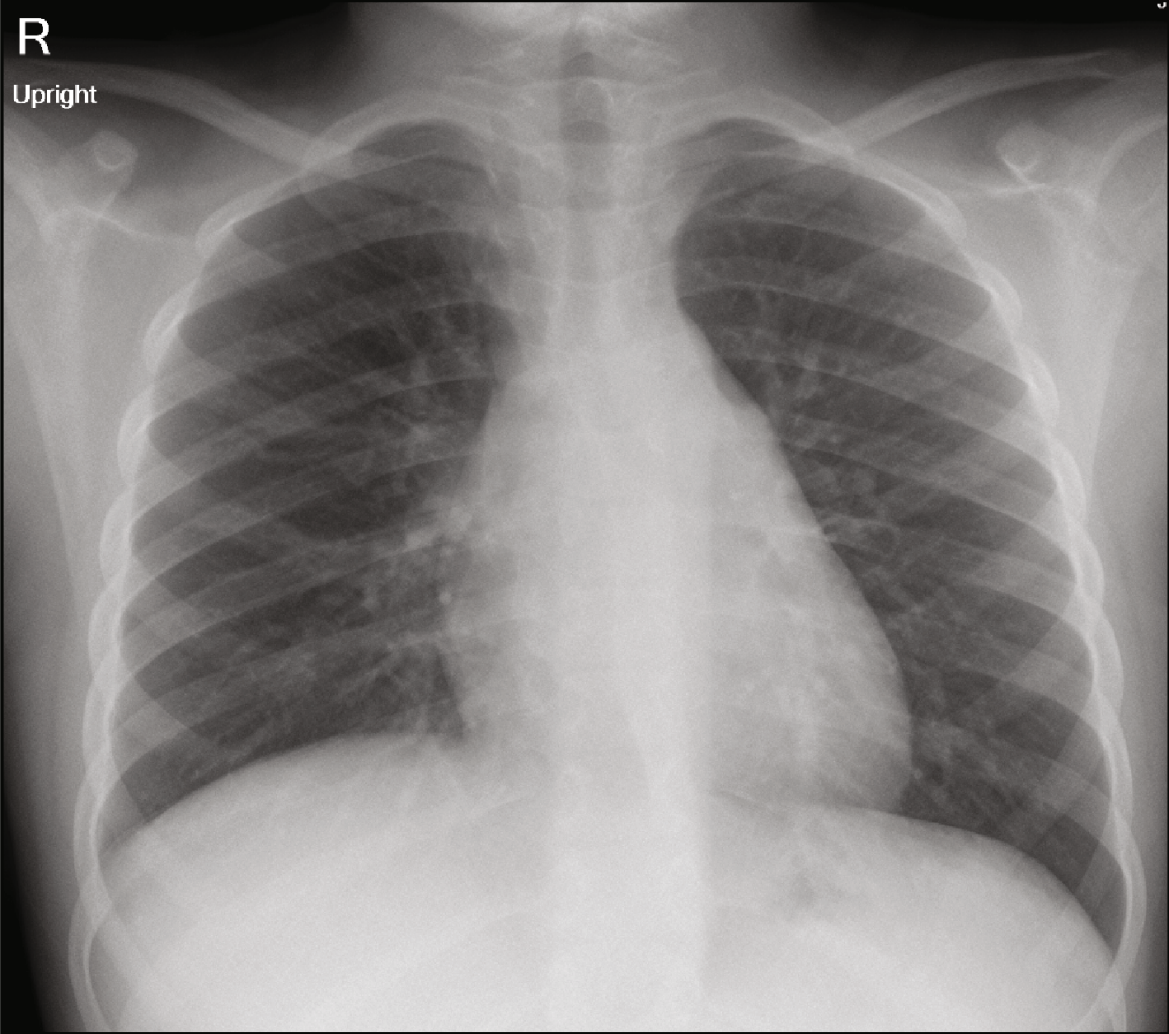
PA view of chest X-ray at the 4-month follow-up visit: Both sides of the lungs were well expanded and clear, with no residual changes noted in the middle lobe. There was no pleural effusion, only trace thickening at the right basal pleura.

The patient received IV antibiotics upon admission, but they were discontinued as soon as genetic testing confirmed the FMF diagnosis and ruled out infections. He was then started on oral colchicine at a dose of 1 mg daily, which significantly alleviated his chest pain. Subsequently, he was discharged home to continue therapy. During follow-up clinic visits 4 months and 6 months later, he reported no chest pain or fever. A repeat chest X-ray showed complete resolution of the right-sided pleural effusion with residual right basal pleural thickening (Figure 3).

The patient continued to be followed by the GI team for his CD, and he was maintained on adalimumab subcutaneous injections every 2 weeks besides colchicine therapy.

The patient's family was referred for genetic counseling regarding parental testing and family planning. However, the parents were unable to proceed with testing due to financial limitations.

## 3. Discussion

FMF and CD share similar clinical and biological features, allowing for the possibility of co-occurrence [8]. This can sometimes make the diagnosis of either illness or both challenging. Though the occurrence of both conditions in the same patient is uncommon, our case, along with previously reported cases, demonstrates that it can occur [9].

CD is a complex condition influenced by genetic and environmental factors. While genetic testing, including screening for *NOD2* mutations, is not routinely used for CD diagnosis, it can be valuable in research or atypical cases, aiding in the identification of pathogenic variants and potential risk factors [10]. In our case, CD was diagnosed based on clinical symptoms, endoscopic and histopathological findings, laboratory markers, and response to treatment. Genetic testing for early-onset IBD was also performed but yielded negative results.

The patient later experienced recurrent fever and chest pain for over a year, which were initially attributed to recurrent infections due to immunosuppression. Ultimately, the diagnosis of FMF was made, highlighting the challenge of distinguishing between the overlapping symptoms of CD and FMF.

Epidemiological studies have shown that the frequency of IBD can be high in patients with FMF [5, 11]. The co-localization of the pyrin and *NALP3* genes (*MEFV* and *NLRP3*, respectively), both of which are involved in inflammatory signaling pathways, indicates that *MEFV* is also a potential IBD gene [12]. *MEFV* gene mutations can possibly trigger or accentuate the inflammatory condition of IBD patients or vice versa [13].

This shared mechanism is further supported by several epidemiological studies that found a high number of *MEFV* gene mutations in patients with IBD compared to the general population. Beşer et al. found that 26.4% of the 53 children with IBD had FMF [14]. Urgancı et al. revealed that 41.9% of 597 children with IBD had FMF mutations [15]. Published studies are inconsistent, however, regarding their descriptions of the course of the disease in patients suffering from both FMF and CD (FMF–CD phenotype) compared to those with either CD or FMF alone. Some studies found that the IBD–FMF group has more severe IBD and experiences more FMF attacks when compared to those with IBD and FMF alone, respectively [14]. Other studies were unable to replicate these findings [3, 5].

Several MEFV variants, including *M694V*, *V726A*, *M680I*, *M694I*, and *E148Q*, are among the most frequently identified in FMF and are known to impact disease severity. However, other pathogenic *MEFV* variants are also linked to diverse clinical presentations and varying degrees of disease severity [16, 17]. These genotypic insights enhance FMF diagnosis, prognosis, and treatment. Moreover, the prevalence of specific *MEFV* variants varies significantly among different populations and geographic regions. In Lebanon, the largest study conducted on FMF found a high frequency of *M694V* and *V726A* mutations, both of which were linked to more severe disease compared to the homozygous *E148Q* genotype [18]. Conversely, in a cohort of Egyptian patients with genetically confirmed FMF, *E148Q* was identified as the most common mutation (31%), followed by *M680I* (G/A), which showed the strongest association with elevated serum amyloid A levels [19].

While CD primarily affects the gastrointestinal system and FMF mainly involves serosal inflammation, including pleurisy and pleural effusion, the overlap in their pulmonary and gastrointestinal manifestations can make diagnosing either condition, or both, particularly challenging.

Pulmonary symptoms in patients with IBD are uncommon and can include airway inflammation, interstitial lung disease, and serositis. Pleural effusion is rare, typically unilateral, and exudative in nature [20]. Conversely, pleurisy ranks among the three most common symptoms of FMF, alongside abdominal pain and arthritis [2]. Pleurisy and pleural effusion are not typical manifestations of CD; therefore, their presence should raise suspicion for FMF diagnosis, as ultimately occurred in our patient.

Gastrointestinal manifestation of FMF, on the other hand, is primarily peritonitis [2]. Endoscopic and pathological findings are rare in FMF. However, recent reports have shown that FMF can affect the colon and small intestine [21]. Endoscopic findings of mucosal erythema, erosion, and ulceration have been reported [22].

Our patient had a positive family history of FMF, which also aided in reaching the FMF diagnosis as a concomitant disease to CD. Genetic testing revealed heterozygous mutations *M680I* (G/A) and *M694I*.

One limitation of this case is the lack of parental carrier testing to determine if the two identified *MEFV* pathogenic variants are on the same allele (cis) or separate alleles (trans). This distinction is crucial for confirming a molecular diagnosis of FMF, which requires biallelic pathogenic variants to be in trans [23]. Without segregation analysis, it remains uncertain whether the patient is a compound heterozygote or if both variants reside on a single allele, complicating molecular confirmation as observed in some clinically diagnosed FMF patients [24]. Parental testing was not conducted due to cost limitations and family preferences, introducing uncertainty about the variants' zygosity. If the variants are in cis, the molecular diagnosis would not be confirmed; conversely, if they are in trans, the diagnosis would be validated. Nonetheless, our patient's clinical presentation and positive response to colchicine support the diagnosis. Future cases would benefit from parental testing to ensure more accurate genetic interpretation and reinforce the molecular diagnosis of FMF.

*MEFV* gene testing is now the primary diagnostic test for FMF and should be considered in patients with IBD who exhibit suspicious symptoms of FMF. In our case, the FMF diagnosis was delayed due to the assumption that the patient's pulmonary symptoms and chest X-ray findings were a result of recurrent lower respiratory tract infections in an immunosuppressed patient. Awareness of the symptom overlap and the high concomitant rate between IBD and FMF helps prevent such delays.

The main goal of treating both FMF and CD is to control disease activity. Colchicine is the primary treatment for FMF, effectively managing symptoms and preventing amyloidosis, a known long-term complication of FMF [25]. In contrast, the management of CD relies on biological agents and immunosuppressants tailored to disease severity and patient response [26, 27]. Our patient was treated with colchicine for FMF alongside treatment for CD, achieving good control of both conditions.

In conclusion, it is essential to consider the likelihood of FMF in patients with IBD who have suspicious respiratory symptoms as a concomitant disease to ensure early diagnosis and treatment and to prevent long-term complications.

## Data Availability

The data that support the findings of this study are available from the corresponding author upon reasonable request.
